# Human Lung Mononuclear Phagocytes in Health and Disease

**DOI:** 10.3389/fimmu.2017.00499

**Published:** 2017-05-01

**Authors:** Faezzah Baharom, Gregory Rankin, Anders Blomberg, Anna Smed-Sörensen

**Affiliations:** ^1^Immunology and Allergy Unit, Department of Medicine Solna, Karolinska Institutet, Karolinska University Hospital Solna, Stockholm, Sweden; ^2^Department of Public Health and Clinical Medicine, Division of Medicine, Umeå University, Umeå, Sweden

**Keywords:** respiratory, pulmonary, monocytes, dendritic cells, macrophages, bronchoalveolar lavage, lung tissue, bronchial tissue

## Abstract

The lungs are vulnerable to attack by respiratory insults such as toxins, allergens, and pathogens, given their continuous exposure to the air we breathe. Our immune system has evolved to provide protection against an array of potential threats without causing collateral damage to the lung tissue. In order to swiftly detect invading pathogens, monocytes, macrophages, and dendritic cells (DCs)—together termed mononuclear phagocytes (MNPs)—line the respiratory tract with the key task of surveying the lung microenvironment in order to discriminate between harmless and harmful antigens and initiate immune responses when necessary. Each cell type excels at specific tasks: monocytes produce large amounts of cytokines, macrophages are highly phagocytic, whereas DCs excel at activating naïve T cells. Extensive studies in murine models have established a division of labor between the different populations of MNPs at steady state and during infection or inflammation. However, a translation of important findings in mice is only beginning to be explored in humans, given the challenge of working with rare cells in inaccessible human tissues. Important progress has been made in recent years on the phenotype and function of human lung MNPs. In addition to a substantial population of alveolar macrophages, three subsets of DCs have been identified in the human airways at steady state. More recently, monocyte-derived cells have also been described in healthy human lungs. Depending on the source of samples, such as lung tissue resections or bronchoalveolar lavage, the specific subsets of MNPs recovered may differ. This review provides an update on existing studies investigating human respiratory MNP populations during health and disease. Often, inflammatory MNPs are found to accumulate in the lungs of patients with pulmonary conditions. In respiratory infections or inflammatory diseases, this may contribute to disease severity, but in cancer patients this may improve clinical outcomes. By expanding on this knowledge, specific lung MNPs may be targeted or modulated in order to attain favorable responses that can improve preventive or treatment strategies against respiratory infections, lung cancer, or lung inflammatory diseases.

## Introduction

Respiratory diseases are among the leading causes of death worldwide, with lung infections, lung cancer, and chronic obstructive pulmonary disease (COPD) together accounting for several million deaths annually (1). The human respiratory tract can be broadly divided into the upper and lower airways. The upper airways consist of the nose, the pharynx, and the larynx. Organized like a tree, the lower airways begin with the trachea branching out into the bronchi, the bronchioles, and eventually the alveoli. A dense network of capillaries underlying the alveoli forms the basis of respiration with the critical exchange of oxygen and carbon dioxide that is necessary for life. The thin, permeable membrane of the alveolar epithelium is vulnerable to penetration of foreign particles and disruption upon inflammation. In humans, the total alveolar surface area is approximately 140 m^2^ (2). To ensure that such a vast area is well monitored, monocytes, macrophages, and dendritic cells (DCs) serve as sentinels at the interface between the external environment and our body. Together termed mononuclear phagocytes (MNPs), they are a heterogeneous population of antigen-presenting cells that have been well described in the lungs of mice and rats at steady state (3–8). Additionally, blood monocytes infiltrate the lungs upon inflammation (9). Their heterogeneity extends beyond morphological or phenotypic characteristics as different mouse MNPs exert different functionalities (10–12). MNPs play a central role in immune surveillance by being adept at capturing antigen to destroy them or to present them in order to activate the adaptive immune responses (13, 14). However, as shown in mice, each subset excels at different aspects of antigen uptake, processing, and presentation, with distinct capacities to migrate and polarize T cells, hence skewing immune responses differently (15–18). Importantly, investigations into MNPs are shifting toward tissue specificity, as immune cells residing in peripheral tissues have distinct traits compared to those in circulation, due to the cues given by the local microenvironment (19–21). The capacity of lung MNPs to regulate immunity has made them attractive targets for preventive or treatment strategies against respiratory infections, lung cancer, or lung inflammatory diseases (22–24). Before applying our knowledge of lung MNPs to the development of vaccine or therapeutic strategies for patients, a thorough characterization of human lung MNPs is required, to ensure a functional alignment of lung DCs from mice and men.

## Distinct Origins of Monocytes, DCs, and Macrophages

The term MNPs was first coined in the 1960s by van Furth, referring to both circulating monocytes and tissue macrophages (25), as opposed to polymorphonuclear phagocytes (granulocytes) (26), but their history dates further back. In the 1880s, the concept of phagocytosis (from ancient Greek, meaning “to devour”) was established by the Nobel Laureate Elie Metchnikoff, who described the ability of macrophages to engulf foreign entities as a defense mechanism (27, 28). Following labeling studies using radioactive thymidine, monocytes were defined as precursors of macrophages circulating in blood, as extensively studied by van Furth and others. In 1973, the Nobel Laureate Ralph Steinman discovered a novel type of “dendritic-shaped cell that can process and present antigen to activate naïve T cells” in the spleen of mice, calling them DCs in his seminal papers (29–32). DCs then joined monocytes and macrophages as another member of the MNP system.

**Figure 1:**
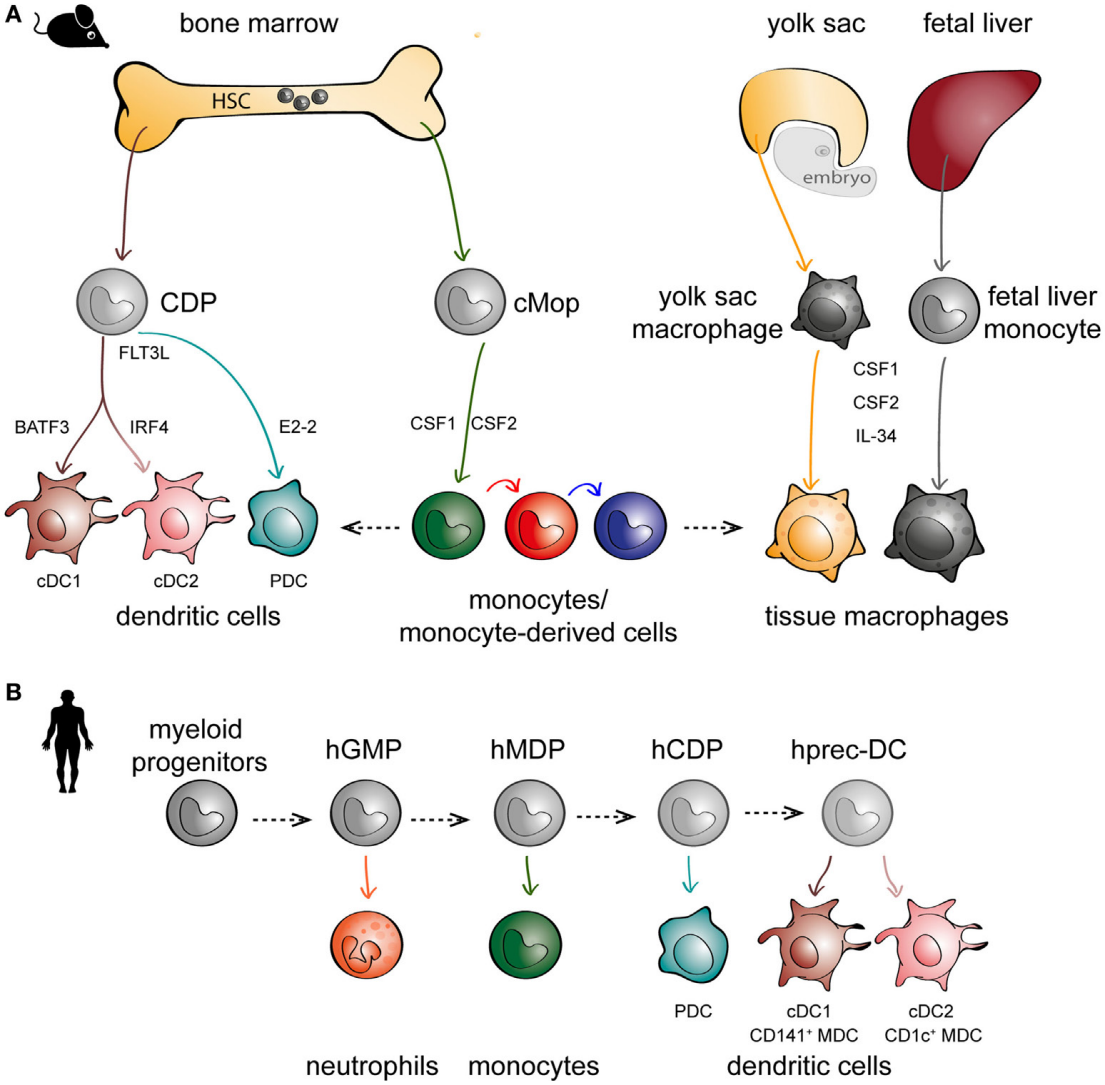
**The embryonic and hematopoietic development of mononuclear phagocytes (MNPs) in mice and men**. **(A)** In mice, monocytes, dendritic cells (DCs), and macrophages originate from distinct lineages. DCs (left panel) and monocytes (middle panel) originate from hematopoietic stem cell (HSC) precursors known as committed DC progenitor (CDP) and common monocyte progenitor (cMop), respectively. Some tissue macrophages have an embryonic origin, either from yolk sac macrophages or fetal liver monocytes (right panel). DCs express different transcription factors critical to their development, such as basic leucine zipper ATF-like 3 (BATF3) for cDC1s, interferon regulatory factor 4 (IRF4) for cDC2s, and E2-2 for plasmacytoid DCs (PDCs). MNPs are also differentially dependent on various growth factors such as Fms-related tyrosine kinase 3 ligand (FLT3L) for DCs (left panel), CSF1 and CSF2 for monocytes (middle panel), and colony-stimulating factor 1 (CSF1) and CSF2 and IL-34 for macrophages (right panel). **(B)** In humans, *in vitro* culture models have been used to recapitulate *in vivo* DC hematopoiesis employing progenitors from human cord blood and bone marrow; hGMP, human granulocyte–monocyte–DC progenitor; hMDP, human monocyte–DC progenitor; hCDP, human common DC progenitor; hprec-DC, human migratory precursor.

For decades since their discovery, monocytes were thought to only exist in peripheral blood, differentiating into DCs and macrophages upon entry into tissues (33). This was supported by the ease in which monocytes can be skewed to behave like DCs or macrophages *in vitro* (34–37), depending on the culture conditions, and also *in vivo* during inflammation (38–41). However, more careful lineage studies in mice have identified hematopoietic precursors to DCs [called committed DC progenitors (CDPs)] that are distinct from monocytes (Figure 1A, left panel) (42–44). CDPs can further differentiate to plasmacytoid DCs (PDCs) or pre-classical DCs (cDCs) that can become cDC1 [CD141^+^ myeloid DCs (MDCs) in humans] or cDC2 (CD1c^+^ MDCs in humans). Maintenance of DC development is linked to their expression of Fms-like tyrosine kinase 3 (FLT3) and their ability to respond to FLT3 ligand (45, 46). In parallel, monocytes diverge at an earlier stage and are derived from a different progenitor (called common monocyte progenitor) (47), relying on the cytokine colony-stimulating factor 1 for their development (Figure 1A, middle panel). These observations in mice have also been confirmed in humans following the identification of DC precursors in circulation, cord blood, and bone marrow (Figure 1B) (48, 49). Another paradigm-shifting discovery is that tissue-resident macrophages are not exclusively derived from circulating monocytes, as has been the dogma following van Furth’s findings in the 1960s. Instead, mouse tissue-resident macrophages can develop from embryonic precursors such as yolk sac macrophages or fetal liver monocytes (5, 50–56) (Figure 1A, right panel). In short, there is mounting evidence to suggest that monocytes, DCs, and macrophages are not developmental progressions from one cell type to another, but instead originate from distinct precursors.

The matter is complicated by the plasticity of monocytes that can acquire different functional properties shared by macrophages and DCs, depending on the inflammatory environment (57, 58). Identification of cell types based purely on expression of surface markers or functional specialization presents a challenge as several different populations share the same receptors, and subsets can acquire or lose functional capacities during inflammation (59). Beyond semantics, the definition of cell populations is important for interpretation and translation of findings between different groups, especially when specific functional attributes are assigned to distinct populations. A shift toward complementing phenotypic identification with transcriptional profiling has allowed a better separation of DCs, monocytes, and macrophages, including a better alignment of cells across species and tissues.

## Techniques for Sampling the Respiratory Tract of Humans

**Figure 2:**
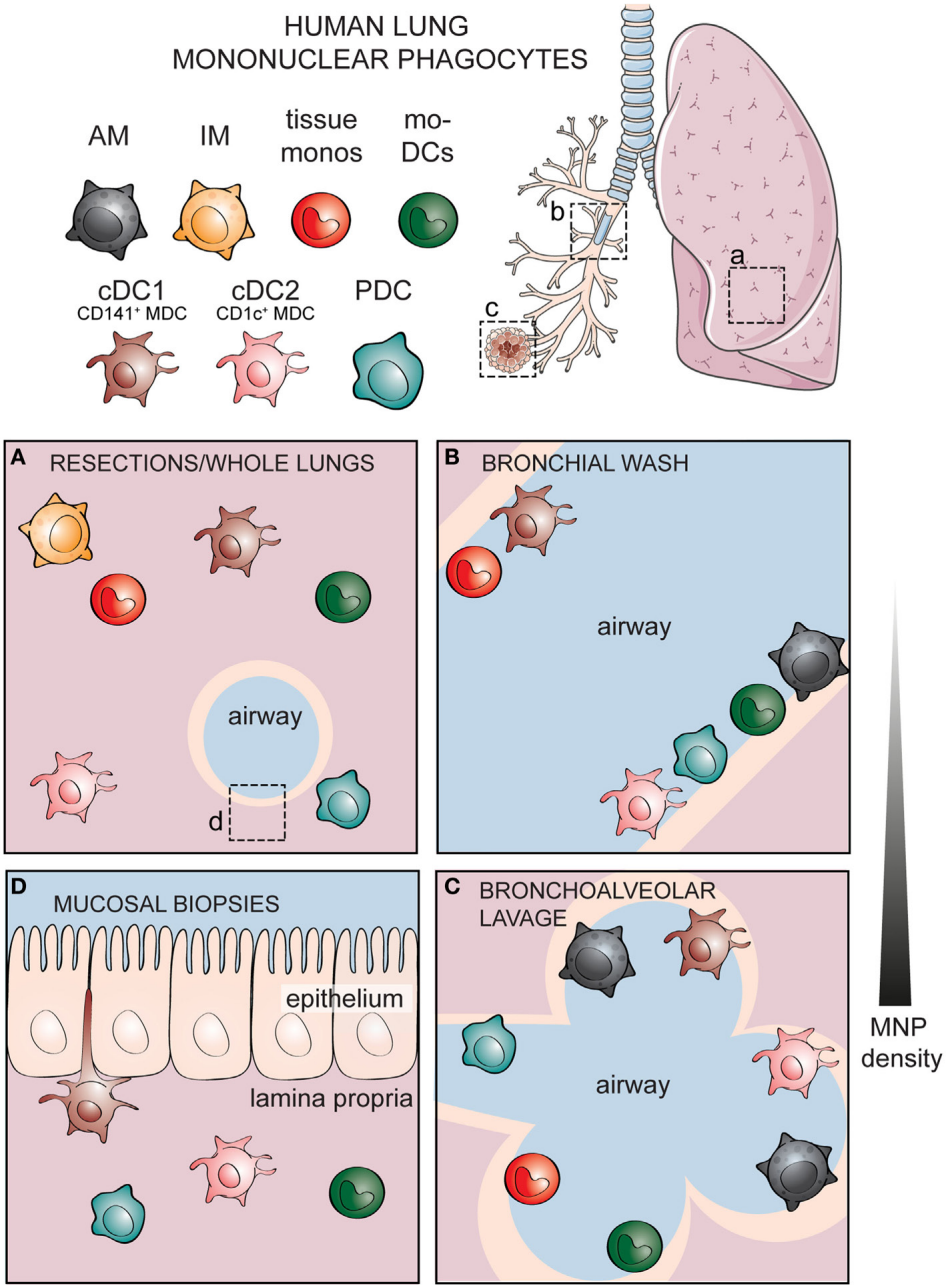
**Identities of human mononuclear phagocytes (MNPs) differ in specific respiratory compartments sampled by different methods**. Alveolar macrophages (AMs), interstitial macrophages (IM), tissue monocytes, monocyte-derived dendritic cells (mo-DCs), and three subsets of *bona fide* dendritic cells including cDC1, cDC2, and plasmacytoid DCs (PDCs) have been documented in different compartments of the human respiratory tract. **(A)** Cells within the lung parenchymal tissue can be assessed in whole lungs or surgical resections. Cellular components of the airways can be sampled by performing lavages including **(B)** a shallow bronchial wash or **(C)** a deeper bronchoalveolar lavage, whereas **(D)** cells within the respiratory mucosal tissue can be sampled by taking mucosal biopsies. Lung illustration modified from Servier Medical Art.

There are several methods of sampling the human respiratory tract. The most common source of human lung tissue comes from surgical resections, due to lung tumor or other lung diseases (Figure 2A) (60). The surrounding, non-diseased parts of the lungs are used in studies as a representation of healthy tissue. These samples constitute parenchymal lung tissue. However, as the lungs are highly vascularized, the surgical tissues obtained also consist of intravascular cells from the circulation. Lungs from organ donors that are available but not used for transplantation are an alternative source of lung tissue for immunological research (61). Perfusion of whole lungs is possible to remove intravascular cells, in order to discriminate between cells residing in the lung tissue and those in the lung vasculature (62). Further, investigation of lung-associated draining lymph nodes is possible with whole lungs, enabling the assessment of migration to lymphoid tissues and interaction with adaptive immune cells—key features of DCs (62, 63). A dependence on surgical lung material or lung transplants presents a challenge to experimental research, as both types of samples are not readily available. Furthermore, migratory immune cells such as MNPs isolated from adjacent sites may already be affected by unpredictable direct or bystander effects due to the diseased tissue. Lung specimens can also be acquired by performing a bronchoscopy to obtain bronchial wash (Figure 2B), bronchoalveolar lavages (Figure 2C), bronchial biopsies (Figure 2D), or bronchial brushing (64–66). Unlike the parenchymal tissue obtained from surgical resections or organ transplants, specimens obtained by bronchoscopy reflect cells lining the airways and those embedded within mucosal surfaces. Sequential lavages allow separation of bronchial and alveolar samples, termed bronchial wash (Figure 2B) and bronchoalveolar lavage, respectively (64). One of the advantages of using cells from lavages is that the cells undergo minimal manipulation prior to phenotypic and functional analyses, unlike tissue samples that need to undergo mechanical processing or enzymatic digestion in order to obtain single cells. An alternative approach to understanding human immune cells in inaccessible tissues is by harnessing the humanized mouse models (67, 68). However, humanized mouse models have their own caveats: the lung architecture of mice is different from that of humans, as murine lungs show less airway branching and lack respiratory bronchioles (69). Importantly, cytokines and chemokines produced by mouse epithelial cells may not act on the human cognate receptors (70). Knock-in mice expressing human cytokines have been developed to address this limitation (67). Since immune cells are in close contact with the respiratory epithelium (71), compromised communication between epithelial cells and immune cells may influence immunological events in humanized mice. Finally, given the massive size of the respiratory tract, the composition of immune cells may not be equally distributed but instead vary depending on which portion of the lungs is being examined. Considering the anatomic compartmentalization within the lungs may be valuable when comparing findings between studies using different sources of respiratory material.

## Phenotypic and Functional Characterization of Human Lung MNPs

Cell surface markers continue to be a reliable source of information for classification of DCs, monocytes, and macrophages based on their phenotype, preferably supported by transcriptomic and functional analyses. Comparative studies have attempted to unify the MNP populations between mice and humans. The cell surface markers used to identify and sort out individual populations of MNPs based on flow cytometry are summarized in Table 1. A common flow cytometric gating strategy used by our group and several others to identify DCs in blood and tissue is by first gating on all hematopoietic cells (CD45^+^), excluding all lineage cells [monocytes, B cells, T cells, natural killer (NK) cells, and neutrophils] and then gating on cells expressing the MHC class II molecule, HLA-DR^+^ cells to identify DCs (72, 73). CD11c can be used to distinguish MDCs from PDCs. Aside from peripheral blood, these DC populations have also been identified in human bone marrow, skin, gut, lungs, liver, spleen, lymph nodes, and tonsils (62, 73–78). However, the precise phenotype of DCs in human tissue continues to be investigated and debated upon, with the most studied tissue in humans being the skin (79, 80). In light of different markers used to identify DCs in different human tissues, Guilliams et al. propose a framework to standardize the identification of DCs in human tissues at steady state and during inflammation (81). Here, we revisit early studies investigating human lung MNPs and review the most recent developments in the field, with special attention to cellular players in specific respiratory compartments.

**Table 1:** **Cell surface markers of mononuclear phagocytes in human lungs**.

Ontogeny	Macrophages	Monocytes			Committed DC progenitor			Reference
Surface marker	Alveolar macrophages	Interstitial macrophages	Tissue monocytes	Monocyte-derived DC	cDC1 CD141^+^ MDC (IRF8 dependent)	cDC2 CD1c^+^ MDC (IRF4 dependent)	PDC	
	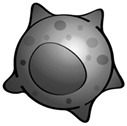	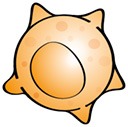	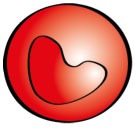	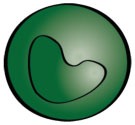	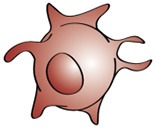	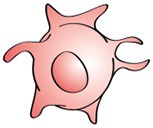	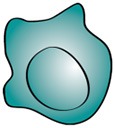	
AF	++	−	−	−	−	−	−	(73, 95)
BTLA	n.d.	n.d.	n.d.	−	++	+	n.d.	(75)
CADM1	n.d.	n.d.	n.d.	n.d.	+	−	n.d.	(81)
CD1a	−	−	−	−/+	−	−/+	−	(62, 73, 74)
CD1c	−	−	−	+	−	+	−	(62, 63, 73–75)
CD11b	+	+	+	+	−	+	−	(62, 75, 81)
CD11c	+	+	+	+	+	+	−	(62, 63, 68, 73–75, 95)
CD14	−	+	+	+	−	−	−	(62, 73, 95)
CD16	+	+	++	−	−	−	−	(62, 73, 93)
CD64	+	+	+	−	+	+	−	(62, 75, 130)
CD103	+	n.d.	−	−	−/+	+	+	(122)
CD123	−	−	−	−/+	−	−/+	+	(73, 93)
CD141	+	−	−	−/+	++	+	−	(63, 73, 74)
CD163	+	+	+	+	−	−	−	(62, 73, 130)
CD169	+	−	n.d.	−	−	−	−	(93)
CD172a	n.d.	n.d.	n.d.	+	−	+	n.d.	(75, 81)
CD206	+	+	+	+	−	+	−	(62, 73, 93)
CD207	n.d.	n.d.	n.d.	n.d.	−	−/+	−	(123)
CD303	−	−	−	−	−	−	+	(73)
Clec9A	−	−	−	−	+	−	−	(74)
HLA-DR	+	+	+	+	++	++	++	(62, 63, 68, 73–75, 81, 93, 95, 122, 123, 130)
Lineage^a^	−	−	−	−	−	−	−	(62, 63, 68, 73–75, 81, 93, 95, 122, 123, 130)
TGFbR	n.d.	n.d.	n.d.	n.d.	+	+	n.d.	(68)
XCR1	−	−	−	−	+	−	−	(74)

*^a^CD3, CD19, CD20, CD56, CD66abce*.

*n.d., not determined; DCs, dendritic cells; PDC, plasmacytoid DC; cDC, classical DC; IRF, interferon regulatory factor; MDC, myeloid DC*.

### Alveolar Macrophages (AMs)

Alveolar macrophages are the first MNP population to be described, given the relative ease of obtaining them by bronchoalveolar lavage and their abundance (up to 95% of cells lavaged in healthy subjects are AMs) (82). AMs play an important role in removing surfactants and other foreign materials, ensuring that the lungs remain free of debris (83). Impaired AM function in patients result in pulmonary alveolar proteinosis, a rare disorder that can be treated with granulocyte/macrophage colony-stimulating factor (84, 85). It was reported in the 1970s that human AMs have local proliferative capacity and therefore need not be replenished by monocytes from the bone marrow (86). Yet, AMs can also be replenished by monocytes from the bone marrow, as illustrated in patients receiving bone marrow transplants for hematologic disorders (87). The seemingly contradictory findings are now better understood based on recent studies in mice indicating that AMs can be derived from yolk sac macrophages, fetal liver, and adult monocytes given a vacant niche (56). In more recent studies involving human lung transplantations, almost 100% of AMs detected in BAL are donor derived, with a capacity to self-renew (88, 89). This finding supports the niche hypothesis, as transplanted lungs already occupied by the donor’s AMs need not be replaced by the recipient’s circulating monocytes (88, 89).

Pioneering studies on human MNPs in the lungs relied mostly on morphology visualized by microscopy and expression of single markers such as CD11c and HLA-DR by immunohistochemistry. By flow cytometry, human AMs can be identified based on their large size and high granularity (90, 91). Additional markers currently used for identification of AMs include CD206, CD163, and CD169, although there may be overlaps with other lung phagocytes (Table 1) (92–94). The superiority of human AMs at internalization of bacterial particles, compared to lung DCs or monocytes was recently highlighted by Patel et al. upon *in vitro* exposure of lung phagocytes to different bacteria such as *Escherichia coli, Staphylococcus aureus*, and *Bacillus anthracis* (95). However, unlike human lung DCs or monocytes, AMs do not upregulate CD83, CD86, or CCR7 upon exposure to bacteria (95). Despite their surface expression of HLA-DR, AMs are poor inducers of antigen-induced T cell proliferation and also poor stimulators of allogeneic mixed lymphocyte reactions (MLR) (96–98). Instead, AMs promote tolerance by suppressing lymphocyte activation *via* production of transforming growth factor β (TGF-β) and prostaglandins (99, 100). Although AMs are typically quiescent in order to minimize damage to the delicate alveoli, they can mount inflammatory responses when necessary (101). They release soluble mediators such as IL-8, a chemotactic factor important in the recruitment of neutrophils into the airways (102–104). Transcriptomic profiling of human AMs upon LPS stimulation has identified interferon-related genes that can fine-tune the early cytokine responses (105). The dynamic roles of AMs will be further discussed in this review during different disease conditions.

### Dendritic Cells

Dendritic cells have been described in the nasal mucosa (106–108), the epithelium, and submucosa of conducting airways (6, 7, 109, 110), the lung parenchyma (7), and also alveolar surfaces (91). The first reference to human lung DCs was in 1986, when Sertl et al. identified HLA-DR^+^ cells in preparations of human airway epithelium, lung parenchyma, and visceral pleura by light and electron microscopy (7). HLA-DR^+^ DCs with extending processes are interspersed between columnar epithelial cells in the large airways; in samples obtained from the lower trachea and mainstem bronchi. Lung mononuclear cells isolated from whole lungs are able to stimulate T cells in an allogeneic MLR more efficiently than blood monocytes (111). Removal of FcR^+^ cells improved the efficiency of stimulating allogeneic T cells, suggesting that the lung mononuclear cells consist of both DCs and monocytes/monocyte-derived cells (111). However, compared to blood monocytes, lung DCs are less potent at producing pro-inflammatory cytokines such as interleukin 1 (IL-1) and tumor necrosis factor (TNF) in response to LPS (112). In bronchoalveolar lavage, mononuclear cells can be distinguished from the majority population of AMs by flow cytometry based on their lack of autofluorescence (73, 90, 91, 113). Immunohistochemical staining *in situ* suggests a local interaction between lung DCs and T cells, as they form small clusters in the subepithelial tissue of the bronchus (91). *In vitro*, the non-autofluorescent mononuclear cells excel at stimulating T cell proliferation, compared to the highly autofluorescent AMs (90, 114, 115). Several differences in the expression of cell surface receptors allow a distinction between lung DCs and blood DCs: a portion of DCs residing in the bronchial epithelium express CD1a (110), and mannose receptor (CD206), as illustrated by their capacity to take-up soluble antigens (114). Due to the limited use of phenotypic markers for identification of lung MNPs, lung DCs referred to in earlier studies may be a mixed population of *bona fide* DCs and monocyte-derived cells. Furthermore, the isolation methods used are often extensive, involving enzymatic digestion followed by Ficoll separation and plastic adherence (114), which may lead to the induction of phenotypical and functional differences.

Following the identification of specific blood DC antigen (BDCA) markers (116), similar subpopulations of human DCs have been detected in the nasal mucosa, lung parenchyma, vascular walls as well as bronchial and alveolar surfaces; PDCs expressing BDCA-2 and BDCA-4 or MDCs expressing either BDCA-1 (CD1c^+^ MDCs or cDC2) or BDCA-3 (CD141^+^ MDCs or cDC1) (62, 68, 73–75, 93, 117–121) (Figure 2). Additionally, DCs expressing CD1a can be detected in both the epithelium and submucosa of the airways (117, 118, 122). More detailed phenotypic analyses revealed that a subpopulation of CD1c^+^ MDCs express CD1a, mannose receptor (CD206) and langerin (CD207) in human lungs, unlike blood CD1c^+^ MDCs (62, 73, 74, 123). Similar to their blood counterparts, lung CD141^+^ MDCs also express CLEC9A, whereas lung PDCs express CD123 (68, 73, 74). Many of the markers mentioned above are unique to humans. However, CADM1 and CD172a, commonly expressed in different tissues of mice and men, can be used to identify cDC1 (CD141^+^ MDCs) and cDC2 (CD1c^+^ MDCs), respectively, thus allowing comparisons of DC subsets across species and tissues (65). Additionally, identification of DC subsets *via* transcription factors such as IRF8 and IRF4, required for the development or function of cDC1 and cDC2, respectively (124), has been successfully used in identifying human lung DCs too (63, 81). This standardization will be valuable in streamlining investigations of the same cell from a specific lineage that may express distinct markers under different conditions.

Importantly, there are functional differences between the different subsets of lung DCs. First, they express different repertoires of toll-like receptors, enabling them to respond to different microbial products. CD1c^+^ MDCs and CD141^+^ MDCs express TLR1, TLR2, TLR3, TLR4, TLR6, and TLR8 at the mRNA level, whereas PDCs express TLR7 and TLR9 (125). This is consistent with the TLR repertoire of the corresponding subsets in blood (126). Upon stimulation of TLR4 with LPS and TLR3 with poly(I:C), both lung CD141^+^ MDCs and CD1c^+^ MDCs induce TNF, IL-1β, and IL-6, whereas stimulation of TLR7/8 induces pro-inflammatory cytokines on lung PDCs (73, 125). Second, they have different T cell stimulatory capacities. Altogether, lung DCs are superior at stimulating allogeneic MLR compared to the highly autofluorescent AMs or lung CD14^+^ monocytes (62, 117, 118). Among the lung DC subsets, CD1c^+^ MDCs are best at activating T cells, followed by CD141^+^ MDCs with a more intermediate capacity, and PDCs being the poorest inducers of T cell proliferation in an allogeneic MLR, when compared side-by-side (125). Further, CD4^+^ T cells cocultured with lung MDCs upregulate the activation marker CD25 compared to unstimulated T cells and can also differentiate into memory T cells by upregulating expression of CD45RO (119). Human lung CD1c^+^ MDCs are potent at inducing a Th17 phenotype upon *Aspergillus fumigatus* challenge by producing IL-23p19, compared to CD141^+^ MDCs or CD14^+^ cells in the lungs (75). Further, human lung CD1c^+^ MDCs induce CD103 expression on CD8 T cells, a marker of tissue residency, *via* expression of TGF-β, unlike CD141^+^ MDCs (68). Following exposure to live-attenuated influenza virus (LAIV) *in vitro*, human lung CD141^+^ MDCs can induce both Th1 (IFN-γ) and Th2 (IL-4) responses (127). Interestingly, lung CD141^+^ MDCs are more efficient than lung CD1c^+^ MDCs at inducing Th2 responses, due to increased expression of OX40 ligand upon exposure to LAIV (127). However, most studies investigating T cell activating capacity of human lung DCs have been conducted *in vitro*. Assessment of lung-associated lymph nodes suggests that only CD1c^+^ MDCs (cDC2) accumulate in follicular zones of lung-draining lymph nodes (62, 63).

### Monocytes and Monocyte-Derived Cells

In human peripheral blood, monocytes can be subdivided into three populations based on their expression of CD14 and CD16: classical monocytes (CD14^+^CD16^−^), intermediate monocytes (CD14^+^CD16^+^), and non-classical monocytes (CD14^dim^CD16^+^). By comparison, less is known about monocytes in the lungs, as they were long thought to differentiate into DCs or macrophages upon arrival into peripheral tissues (9, 25). Only recently have tissue monocytes been demonstrated in peripheral tissues including lungs of mice, deriving from Ly6C^hi^ classical monocytes (5, 12). In human lungs, Schlitzer et al. and Haniffa et al. refer to CD14^+^ cells as CD14^+^ DCs (74, 75), whereas Yu et al. could identify a large population of CD14^+^ cells that express HLA-DR and CD11c but had not characterized them further as they were not the focus of their study (68). Applying a similar flow cytometric gating strategy as used for blood monocytes, we and others have identified three populations of monocytes in lung tissue and bronchoalveolar lavage at varying proportions (62, 73, 93). In the airways, cells corresponding to intermediate monocytes are the most frequent monocyte subset (73). All monocyte populations from the airways responded by producing TNF upon stimulation of TLR3, TLR4, and TLR7/8 *ex vivo* (73). Lung CD14^+^ cells could also stimulate both CD4 and CD8 T cells in MLRs, but not as potently as CD1c^+^ MDCs (62). Desch et al. further subdivided CD14^+^ cells into tissue monocytes and monocyte-derived DCs based on expression of CD1c (62). We could confirm that a subpopulation of monocytes upregulated CD1c in the airways and in bronchial tissue, without upregulating typical macrophage markers (73). In contrast, CD14^+^ cells in the human dermis are referred to as monocyte-derived macrophages, as their gene expression program strongly overlaps with human monocytes and macrophages, but not with human DCs (128). The characterization of interstitial macrophages (IM) in human lung parenchymal tissue has been limited (129). Based on studies in rodents, IM originate from bone marrow-derived monocytes and unlike AMs, have a short half-life (130, 131). As a consequence, they may share common phenotypic markers with monocytes. In rhesus macaques, IM share many cell surface markers as blood CD14^+^ monocytes using an extensive flow cytometric panel, except for CCR2, a tissue-homing chemokine receptor that is not expressed by IM, presumably downregulated by their monocytic precursors upon entering tissue (130). Yu et al. propose the usage of sialoadhesin (CD169) to distinguish between AMs (CD169^+^) and IM (CD169^−^) (93, 94). Taken together, our detailed knowledge on human MNPs during steady state has greatly improved in recent years. Nevertheless, many questions remain, including the extent to which functional qualities of these subsets are cell-intrinsic or influenced by the environment. Investigation of human lung MNPs during respiratory infection or inflammation offers an insight into how these cells behave during dysregulated situations.

## Lung MNPs in Respiratory Diseases

At steady state, monocytes, macrophages, and DCs are important in maintaining homeostasis and ensuring tolerance toward harmless antigens arriving in the lungs. In the event of infection or inflammation, how lung MNPs behave in a perturbed environment may clarify the individual roles the different subsets play in initiating immunity or contributing to pathogenicity. Respiratory diseases encompass several pathological conditions affecting the lungs. Together, lung diseases remain among the leading causes of death and disability (1). Focusing on diseases of the lower respiratory tract, respiratory diseases can be further subdivided into bronchial (e.g., acute bronchitis, COPD, and asthma) or interstitial [e.g., sarcoidosis and idiopathic pulmonary fibrosis (IPF)] diseases (132–134). Bronchial diseases are often obstructive leading to blocked airways, whereas interstitial diseases are restrictive leading to decreased lung volume. Infections with viruses or bacteria can affect both bronchial and interstitial compartments. Lung MNPs are also implicated in lung cancers, divided into small-cell lung carcinoma or non-small-cell lung carcinoma. Here, we discuss the existing literature on the involvement of human lung MNPs in the lung diseases that have been best studied (Table 2). However, care should be taken in prescribing function to specific populations in pathological conditions, as the distinction between monocytes, DCs, and individual subsets within them are limited in earlier studies.

**Table 2:** **Dysregulations in frequencies or functions of lung mononuclear phagocytes in human disease.^a^**

Disease	Cells investigated	Study setup	Observations	Reference
COPD	AMs	88 COPD patients underwent BAL (59 non-exacerbation-prone, 29 exacerbation-prone). AMs challenged *in vitro* with bacteria and TLR ligands.	AMs of exacerbation-prone COPD patients exhibit exhaustion. Lower production of IL-8 and TNF upon bacterial exposure.	(151)

COPD	Langerin^+^ DCs (most likely cDC2)	14 never smokers, 15 smokers without COPD, and 44 COPD patients underwent surgery due to cancer.	Increased number of DCs in airways of patients correlate with severity of disease. CCL20 increased in lungs of COPD patients, implicated in recruitment of CCR6^+^ DCs.	(152)

COPD	cDC1 and cDC2	3 never smokers, 11 smokers without COPD, and 28 COPD patients underwent surgery due to lung volume reduction, pulmonary nodules, or lung transplantation.	Increased expression of co-stimulatory molecules correlate with severity of disease as assessed by GOLD stages.	(154)

COPD	cDC2	7 never smokers, 44 smokers without COPD, and 41 COPD patients underwent surgery. 13 never smokers, 12 smokers without COPD, and 19 COPD patients underwent BAL before and on day 7 of rhinovirus controlled infection.	cDC2s (CD1c^+^ MDCs) display a semi-mature phenotype and are less responsive to LPS.	(158)

Allergic asthma	cDCs and PDCs	7 patients with allergic asthma were challenged with allergen or saline in different lung segments and underwent bronchoscopies.	Increased number of DCs in airways after allergen challenge in asthma patients.	(168)

IPF, sarcoidosis	AMs	15 patients with IPF and 46 patients with sarcoidosis underwent bronchoscopy for collection of BAL and lung biopsies.	Spontaneous production of CCL18 by BAL cells of patients with pulmonary fibrosis. Supernatants from AMs of patients containing CCL18 induce collagen production by normal lung fibroblasts.	(179)

IPF	cDC2 (single stains of CD1a, CD1c, and CD209 defined as immature DCs, CD83, CD86, and CD208 defined as mature DCs)	12 patients with IPF underwent surgery (either open lung biopsies or lung transplantation). Immunohistochemistry on snap-frozen tissue.	Increased number of immature DCs in lungs of IPF patients, compared to controls. Chemokines CCL17, CCL19, CCL20, CCL21, CCL22, and CXCL12 strongly expressed in fibrotic lungs.	(180)

IPF, sarcoidosis	cDCs and PDCs	10 sarcoidosis patients and 8 IPF patients underwent BAL.	Numbers of DCs in BAL of IPF patients are similar to controls but more immature. Fewer CD1a^+^ DCs (most likely cDC2) in BAL of sarcoidosis patients.	(182)

Tuberculosis	AMs	Patients underwent BAL for diagnostic purposes but were negative for infections or other lung diseases. AMs were isolated by overnight adherence to plastic and infected with mycobacteria *in vitro*.	Infection of AMs *in vitro* with virulent mycobacteria higher levels of TNF than AMs infected with attenuated mycobacteria. Higher TNF production correlates with increased growth rate of mycobacteria.	(192)

Tuberculosis	CD1a^+^ DCs (most likely cDC2)	93 patients positive for *Mycobacterium tuberculosis* underwent BAL.	CD1a^+^ DCs can be identified in BAL of patients, expressing an immature phenotype.	(193)

Influenza, RSV	CD14^+^ monocytes, cDCs, and PDCs	Nasal wash samples were collected from 22 children <36 months, eventually confirmed to be influenza positive.	Increased numbers of monocytes, cDCs, and PDCs in nasal wash of patients with influenza, higher than in patients with RSV. Increased levels of CCL2 (involved in recruitment of monocytes and DCs) in nasal wash of influenza patients.	(199)

Influenza	Monocytes	Nasal swab and nasal wash samples were collected from 56 patients confirmed to be influenza positive.	Non-classical monocytes are inversely correlated with levels of pro-inflammatory cytokines in nasal lavage samples of patients.	(200)

Non-small-cell lung cancer	cDCs (single stains of CD208 defined as mature DCs)	74 patients with non-small-cell lung cancer underwent surgery. Immunohistochemistry was performed on paraffin-embedded tumor biopsies.	Mature DCs are localized in tumor-induced bronchus-associated lymphoid tissue, correlating with improved clinical outcome.	(205)

Non-small-cell lung cancer	cDCs (single stains of CD208 defined as mature DCs)	458 patients with non-small-cell lung cancer underwent surgery. Immunohistochemistry and immunofluorescence were performed on paraffin-embedded tumor specimens.	Mature DCs in tertiary lymphoid structures correlate with an infiltration of CD8^+^ T cells and long-term survival.	(206)

*^a^Selected key papers are included in this table*.

*DCs, dendritic cells; COPD, chronic obstructive pulmonary disease; PDCs, plasmacytoid DCs; cDCs, classical DCs; TNF, tumor necrosis factor; MDCs, myeloid DCs; IPF, idiopathic pulmonary fibrosis; RSV, respiratory syncytial virus; AMs, alveolar macrophages*.

A striking observation in many studies is that cigarette smoking can alter both frequency and function of lung MNPs (135–140). Cigarette smokers have substantially increased numbers of AMs in the lungs, with identical capacity to phagocytose bacteria compared to AMs of non-smokers (141, 142). AMs of smokers have altered metabolic and enzymatic activities (138, 140, 143). Production of cytokines such as IL-1 is also reduced in AMs of smokers (139). Hence, it is important to compare observations in patients with not only age- and sex-matched healthy controls but also with similar smoking status. Other inhaled particles including air pollutants, such as diesel exhaust, however, typically cannot be controlled in study subjects. These inhaled particles have been shown to impair the ability of AMs to phagocytose, described in detail in the following review (144). As such, comparisons of patient groups with endemic healthy controls exposed to similar particulate matter in the air would account for the contribution of environmental factors.

### Chronic Obstructive Pulmonary Disease

Lung MNPs play a central role in COPD, a disease characterized by aberrant inflammatory responses to cigarette smoke and other inhaled particles (145–147). Persistent inflammation occurs in the lungs of COPD patients (148). A key player is the pro-inflammatory cytokine TNF, potentially produced by lung MNPs, which is increased in sputum and serum of COPD patients (149). However, anti-TNF treatment with infliximab alone was not effective on COPD patients (150). The timing of treatment may be an important factor, as AMs in exacerbation-prone COPD patients exhibit exhaustion: upon bacterial challenge *in vitro*, poorer cytokine responses are observed compared to non-exacerbation-prone COPD patients (151). Langerin^+^ DCs expressing CCR6 accumulate in the airways of COPD patients, increasing with disease severity and higher levels of the chemoattractant CCL20 (the ligand for CCR6) (152, 153). Maturation markers such as CD40, CD80, CD83, and CD86 are also upregulated on lung DC subsets in COPD patients, correlating with disease severity (153–155). Conflicting with this observation, others report that in their cohort of COPD patients, DCs are more immature than DCs isolated from smokers without COPD (156, 157). Tsoumakidou et al. support the hypothesis that lung DCs in COPD patients are tolerogenic by reporting that lung CD1c^+^ MDCs produce more IL-10 and induce regulatory T cells, unlike CD1c^+^ MDCs from smokers without COPD (158). Differences in source of lung tissue and sample preparation may contribute to the conflicting data, underlining the importance of harmonized protocols to allow for comparisons across different study cohorts.

### Asthma

Asthma is characterized by bronchial hyperresponsiveness and influx of inflammatory cells, and the role of lung MNPs in asthma has been widely studied (159–161). Lambrecht et al. pioneered the field more than 15 years ago by illustrating how DCs induce Th2 responses to inhaled antigens in mice (162, 163). Indeed, studies in humans indicate that DCs, especially CD1c^+^ MDCs, accumulate in sputum and bronchial mucosa of asthmatic patients upon allergen challenge (164–169). The increase in DCs in the lungs can be controlled by inhaled corticosteroids, currently the preferred treatment for asthma management (164). In pediatric patients with steroid-treated asthma, their airway DCs expressed lower levels of the co-stimulatory molecule CD86 (167). Supporting data in mouse models suggesting that DCs are responsible for maintaining Th2 responses to inhaled allergens (170), Greer et al. report an increase in CD1c^+^ MDCs in the epithelium of patients with high expression of Th2 genes in the airways, but not in those with low Th2 genes (169). In addition to Th2-driven asthma, Th17 cells and cytokines have been described as driving more severe disease with neutrophilic inflammation (171). Although human MNPs have not been investigated in contributing to Th17-mediated asthma, existing studies suggest that CD1c^+^ MDCs can control mucosal IL-17 responses (75). AMs are also implicated in airway remodeling (172), a central feature of asthma. Further, AMs from asthmatic patients appear to overexpress CCL17, the ligand for CCR4 that is upregulated on T cells homing to the lungs (17, 173). This may contribute to airway inflammation experienced by asthmatics.

### Sarcoidosis and IPF

Pulmonary sarcoidosis is characterized by the formation of granulomas in the lungs: T cells accumulate in the lungs surrounding an unknown antigen that has been phagocytosed by AMs or DCs (174). AMs from sarcoidosis patients produce a variety of cytokines including TNF, IL-2, and IL-6 (175). Massive TNF production by AMs is indicative of increased disease progression (176). Pulmonary fibrosis, observed in both IPF and end stage sarcoidosis, is hypothesized to be a consequence of aberrant wound healing; abnormally excessive production of fibrous connective tissue results in fibrosis (177). Rennard et al. illustrated in the 1980s that AMs from patients with IPF and pulmonary sarcoidosis produce 10 times more fibronectin, a chemoattractant for human lung fibroblasts, than AMs from healthy controls (178). Prasse et al. further identified CCL18, a signature cytokine produced by anti-inflammatory macrophages, as the key component perpetuating pulmonary fibrosis by stimulating collagen production by fibroblasts (179).

Similar to COPD and asthma, DCs accumulate in the lungs of patients with IPF and sarcoidosis, in particular CD1c^+^ MDCs (169, 180–185). In IPF patients, DCs aggregate together with infiltrating lymphocytes, forming an organized lymphoid structure, close to fibroblasts that produce chemokines for recruitment of immune cells (186). In an *in vitro* study, Freynet et al. illustrate that lung fibroblasts from IPF patients may modulate DC function by downregulating their capacity to stimulate T cells in an MLR (187). However, as DCs derived from the Mutz-3 myeloid cell line were used in this study, it remains to be assessed if primary lung DCs of IPF patients would respond in a similar manner. In sarcoidosis, the precise role of DCs is still unclear: there is evidence suggesting that lung DCs initiate the inflammatory T cell response (188), whereas others report that DCs in sarcoidosis patients are anergic and less immunostimulatory (182, 189). This may be a consequence of studying patient samples at different times of disease progression and further studies are needed to better dissect this. A more detailed review of MNP involvement in granuloma formation has been discussed by Broos et al. recently (190).

### Respiratory Infections

Despite extensive studies in mice on the role of MNPs in detecting, controlling, and clearing infection in the lungs, investigation of human lung MNPs during respiratory infection has been limited. Most existing studies exploring the role of human MNPs and respiratory pathogens have used blood-derived MNPs exposed to specific pathogens *in vitro*. Most patients with respiratory infections do not typically undergo surgery or bronchoscopy, except for patients with tuberculosis. Bronchoscopies are occasionally performed on patients infected with *Mycobacterium tuberculosis* (*M.tb*) as a diagnostic strategy. Pathogenesis of tuberculosis involves a complex interplay between the bacterium and the host immune response, reviewed in greater detail by Sasindran and Torrelles (191). A key player, AMs can serve as a reservoir for the bacterium (191). When exposed to *M.tb in vitro*, AMs produce the pro-inflammatory cytokine TNF that correlates with the ability of AMs to support bacterial replication (192). An accumulation of immature DCs has also been reported in the airways of tuberculosis patients (193).

Another respiratory infection that is a global public health concern is influenza. In influenza-infected patients and also in participants of human challenge studies with influenza virus, cytokine levels during infection have been reported to increase both in the lungs and in circulation (194–196). As a potential contributor to pro-inflammatory cytokines, both monocytes and DCs have been reported to accumulate in the nasal mucosa of patients infected with influenza virus, at higher levels than in patients with respiratory syncytial virus (RSV) infection (197–200). However, *in vitro* infection of AMs with a highly pathogenic strain of influenza A virus does not cause excessive TNF production (201). In contrast, *in vitro* infection of AMs with RSV leads to production of TNF, IL-6, and IL-8 (202). In summary, our increased appreciation of how MNPs behave differently depending on their anatomical location suggests that earlier observations using blood MNPs may merit revisiting to more accurately understand the role of human lung MNPs, present at the site of infection, during infection with respiratory pathogens.

### Lung Cancer

In lung cancer, various innate and adaptive immune cells are present in the cancer microenvironment (often referred to as the immune contexture), including MNPs (203, 204). The identification of cancer antigens has accelerated the development of antigen-specific immunotherapy targeting specific DCs to enhance the effector immune response, including cytotoxic T lymphocytes (CTLs), NK cells, and macrophages, ultimately responsible for destruction of tumor cells (23). In a study involving 74 patients with non-small-cell lung cancer, DCs are reported in lymphoid structures close to the tumor in pathologic lung biopsies (205). The density of mature DCs in these tumor sites correlate with improved clinical outcome (205). Similarly, another study involving 458 patients found that increased numbers of mature DCs in tumor-associated tertiary lymphoid structures correlated with an infiltration of T cells carrying an effector memory phenotype (206). In both studies, DCs were identified as cells expressing DC-LAMP (CD208) (205, 206). A more detailed phenotypic analysis of these cells would help in identifying and attributing functional qualities based on our existing knowledge from studies of human lung MNPs. In other studies, expression of programmed death-ligand 1 (PD-L1) on DCs found at tumor sites indicates poor prognosis, as lung DCs with high PD-L1 expression can retain their immature status and thus limit the activation of immunity (23, 207, 208). Hence, drugs such as monoclonal antibodies that target PD-L1 on DCs or the receptor PD-1 on T cells may boost immune responses by removing the inhibitory mechanism of DCs on T cells (209).

## Concluding Remarks

Our understanding of human lung MNPs has improved significantly in the past decade, with combined efforts to describe human counterparts to well-described populations in mice. However, differences in tissue sampling, processing protocols, and phenotypic gating strategies may hamper the ability to directly compare findings between research groups. A more collaborative approach, such as an expansion of the Immunological Genome project (210) focusing on human MNPs, could resolve inconsistencies and also provide broader understanding of the transcriptomic profiles of each population. More attention can also be given to monocytes and monocyte-derived cells in the lungs, given their plasticity and wide range of functionalities. An important body of research exists to support the view that lung MNPs are involved in various lung diseases, as illustrated by their overabundance in the lungs. Production of pro-inflammatory cytokines by MNPs contributes to an amplification of the inflammatory response in the lungs, central to the pathology of many lung diseases. Further efforts should focus on mechanisms leading to the aberrant accumulation of inflammatory MNPs in the lungs in order to aid the development of suitable therapeutic strategies. During respiratory infections, a relevant question to address is whether MNPs encountering pathogens, especially those lining the airways, can translocate and migrate to draining lymph nodes in order to participate in the activation and expansion of pathogen-specific T cells. Although tracking of individual cells would be technically difficult to perform in humans, this knowledge may influence the effectiveness of live vaccines that are delivered intranasally. Further, cancer immunotherapies may be enhanced by targeting specific populations of lung MNPs that can activate cancer antigen-specific CTLs and ensure that they home back to the lungs and remain in the tissue. Finally, the contribution of the microbiome has not been considered by most studies, despite indications that even in healthy humans, a diverse community of microbes exists in our lungs (211). A broader and deeper understanding of the complex cellular and molecular mechanisms dictating tissue trafficking and immune activity of human lung MNPs can be pivotal in our fight against respiratory diseases.

## Author Contributions

FB performed literature review and designed figures and tables. FB and AS-S organized and wrote the manuscript. FB, GR, AB, and AS-S edited the manuscript.

## Conflict of Interest Statement

The authors declare that the research was conducted in the absence of any commercial or financial relationships that could be construed as a potential conflict of interest.
